# Restructuring polymers via nanoconfinement and subsequent release

**DOI:** 10.3762/bjoc.8.151

**Published:** 2012-08-16

**Authors:** Alan E Tonelli

**Affiliations:** 1 Fiber & Polymer Science Program, North Carolina State University, Campus Box 8391, Raleigh, NC, 27695-8301, USA

**Keywords:** cyclodextrins, inclusion compounds, nanoconfinement, organization, polymers, properties, release, urea

## Abstract

During the past several years my students and I have been utilizing certain small-molecule hosts to create nanostructured polymers. This is accomplished by first forming noncovalently bonded inclusion complexes (ICs) between these small-molecule hosts and guest polymers, followed by the careful removal of the host crystalline lattice to obtain a coalesced bulk polymer. We have repeatedly observed that such coalesced polymer samples behave distinctly from those produced from their solutions or melts. Coalesced amorphous homopolymers exhibit higher glass-transition temperatures, while crystallizable homopolymers coalesced from their ICs display higher melting and crystallization temperatures, and sometimes different crystalline polymorphs. When ICs are formed with block copolymers or with two or more different homopolymers, the resulting coalesced samples can exhibit intimate mixing between the copolymer blocks, or between entire homopolymer chains. Each of the distinct behaviors observed for polymers coalesced from their ICs is a consequence of the structural organization of the polymer–host-ICs. Polymer chains in host-IC crystals are confined to occupy narrow channels (diameter ~0.5–1.0 nm) formed by the small-molecule hosts around the included guest polymers during IC crystallization. This results in the separation and high extension of the included guest polymer chains, which leads, following the careful removal of the host molecule lattice, to unique behaviors for the bulk coalesced polymer samples. Apparently, substantial degrees of the extended and unentangled natures of the IC-included chains are retained upon coalescence. In this review we summarize the behaviors and uses of coalesced polymers, and attempt to draw conclusions on the relationship between their behavior and the organization/structures/conformations of the constituent polymer chains achieved upon coalescence from their ICs.

## Introduction

The behaviors and properties of polymer materials are closely related to the organizations, structures, and morphologies of their constituent chains, which can be significantly altered during their processing, unlike the case of atomic and small molecule solids. Because the conformations and arrangements of their inherently flexible long chains are amenable to modifications through processing, materials made from the same polymer can behave very distinctly when the means used to process them are also different. For example, gel-spun Spectra poly(ethylene) (PE) fibers are extremely strong in the fiber direction, and may be fabricated into light-weight armor. On the other hand, molded articles, such as melt-blown PE garbage bags, are not nearly as strong, but have a much greater elasticity, even though the same polymer is used in both applications. The differences in their behaviors are a result of the different organizations, structures, and morphologies of their polymer chains, which are produced by the widely different means used to process PE Spectra fibers and garbage bags.

**Figure 1:**
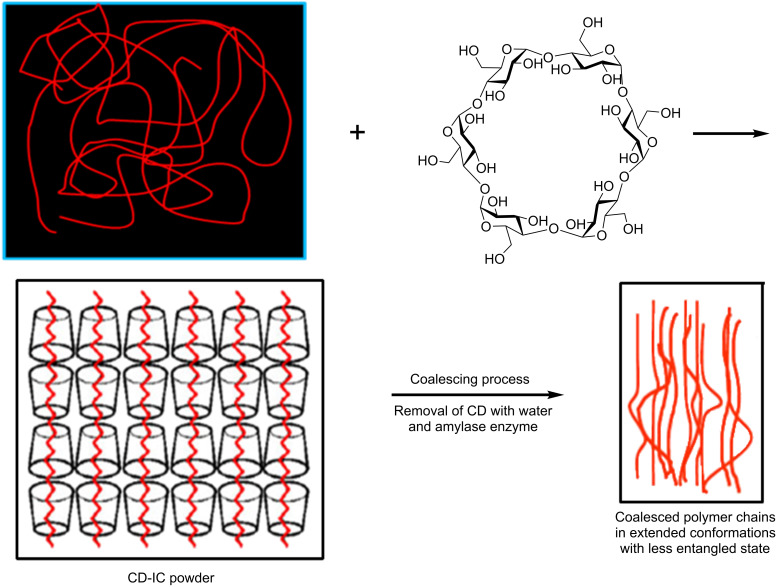
Formation of and coalescence of a polymer sample from its crystalline cyclodextrin inclusion complex [2].

In this review, a means to reorganize polymers by nanoprocessing them into solids with unique properties is presented. This is achieved by first forming noncovalently bonded inclusion complexes (ICs) between certain small-molecule hosts and guest polymers, followed by the careful removal of the host molecules to obtain a coalesced bulk polymer sample. This process is illustrated in Figure 1, in which the cyclic starches, cyclodextrins (CDs), are the host molecules used to form ICs with guest polymers [1–2]. In polymer ICs formed with CDs and other small-molecule hosts, such as urea, thiourea, cyclotriphosphazenes, and perhydrotriphenylenes, the polymer guests are included in very narrow channels (~0.5–1.0 nm in diameter) of the crystalline lattice formed by the host molecules. This results in the isolation and high extension of each included guest polymer chain. By careful removal of the crystalline lattice of host molecules, it was hoped that the resulting coalesced polymer chains (c-polymers) would retain a significant degree of their prior extended and unentangled natures (Figure 1), and thus be organized in a manner quite different from samples processed from their solutions or melts, in which polymer chains randomly coil and entangle. This was indeed found to be the case, and the behaviors and properties of such c-polymer samples were observed to differ significantly from, and to be improved with respect to those of ordinarily processed samples [2–66].

Here, by way of several examples, we attempt to demonstrate the restructuring of polymers by nanoconfinement and subsequent release from their noncovalently bonded ICs. In each case a comparison is made between the behaviors and properties of such c-polymers and samples of the same polymer that were processed in the normal manner. In addition, we demonstrate the use of crystallizable c-polymers to serve as self-nucleants for the melt-crystallization of chemically identical polymers. The behaviors and properties of such self-nucleated polymers are examined and discussed, and their use as reinforcement in the formation of single-component polymer composites is suggested.

## Review

Because the vast majority of the polymer-ICs that we have formed employed CDs as hosts, we herein simply outline the procedures used to form and characterize polymer–CD ICs and to coalesce guest-polymer samples from them. More detailed procedures, as well as the means used to characterize them, may be found in the cited references.

### Formation and characterization of polymer–CD ICs

Polymer–CD ICs are most often produced [2,5–6,17] by combining polymer and CD solutions, usually gradually and with stirring or sonication, followed by filtering off of the resultant IC crystals. These are usually sequentially washed with the same solvents used to make their solutions, to remove any free unthreaded guest polymer and/or host CD, and are then dried.

In some instances, suspension of solid host CDs in polymer solutions or in polymer melts can also lead to IC formation [36,66]. In a related study [67] it was observed that when the α-CD IC containing guest poly(L-lactic acid) (PLLA) chains was suspended overnight in a solution containing poly(ε-caprolactone) (PCL), the resulting solid α-CD IC contained included PCL chains, while the displaced PLLA chains had moved into solution.

**Figure 2:**
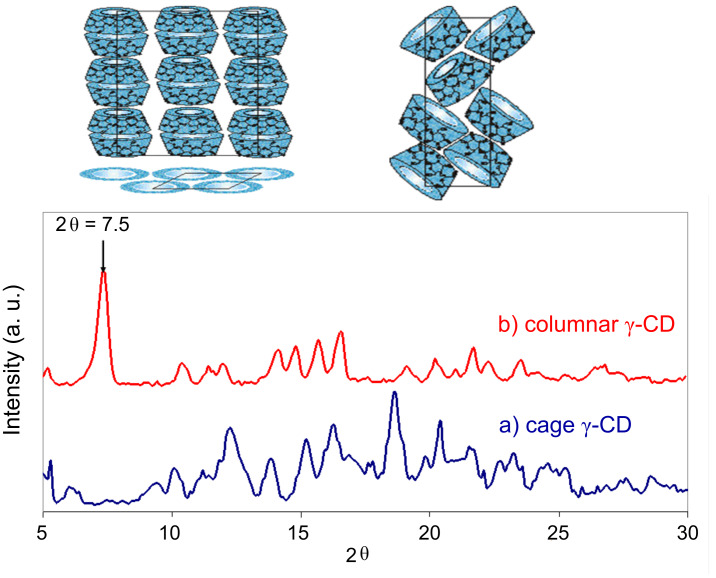
Crystal structures and wide-angle X-ray diffractograms of neat (a) cage and (b) columnar IC γ*-*CD [20].

Polymer–CD ICs are readily characterized by FTIR, NMR, DSC, and X-ray observations [6,8,17,20]. The presence of both guest polymer and host CD can be confirmed by FTIR and NMR spectroscopy, while solid-state ^13^C NMR and X-ray diffraction can confirm the columnar IC structure. For example, in Figure 2 the crystal structures of as-received cage and columnar IC γ-CDs are easily distinguished [20]. Finally, examination by DSC can determine whether the guest polymer has been included in the columnar CD lattice or not, by the absence or presence, respectively, of the thermal signature(s) characteristic of the polymer, i.e., *T*_g_ and/or *T*_m_.

The formation of polymer ICs with host urea (U) and their characterization are similar to those of polymer–CD ICs [69].

### Coalescence and characterization of polymers from their CD ICs

Guest polymers may be coalesced from their CD ICs in several ways [65]. Depending on their mode of formation, they may be washed with warm water, briefly treated with an acidic aqueous solution, or treated with an aqueous solution of an amylase enzyme. Their characterization is accomplished by the same experimental means mentioned above for polymer–CD ICs. In addition, details of the polarizing micrographs, permeabilities, mechanical properties, and rheological behaviors of the c-polymer samples discussed here may be found in references [8,64–65,68–70]. Polymer ICs made with host U are usually coalesced by washing with water and methanol [68].

### Formation and characterization of single-component polymer composites

**Figure 3:**
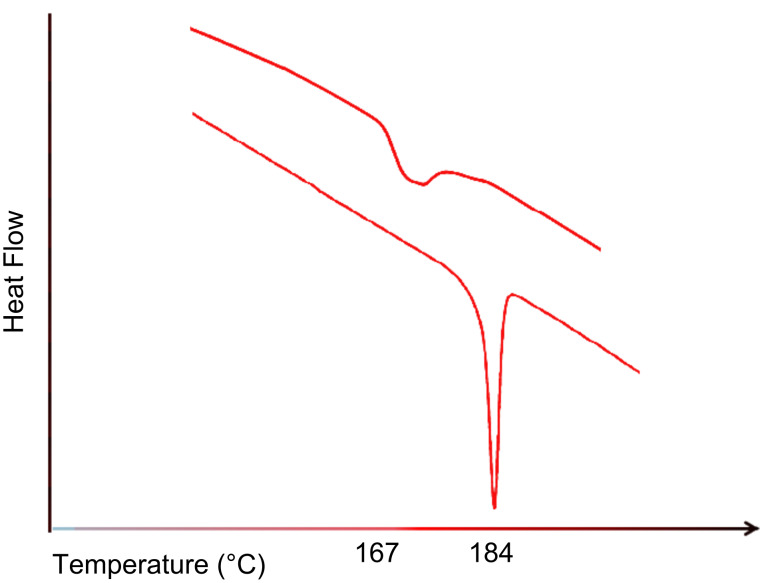
DSC cooling scans of as-received (upper) and coalesced N-6 (lower) [58].

Single-component polymer composites consist of both a matrix and reinforcement made with the same polymer to provide compatible and strong interfaces. This is achieved by forming reinforcing films or fibers with crystallizabilities and mechanical properties superior to those of the matrix they are embedded in. When a crystallizable polymer is coalesced from its CD IC, it is observed to be more readily crystallized upon cooling of its melt [62,64–65], as indicated by a higher crystallization temperature, *T*_c_, and a larger and narrower crystallization exotherm, as can be seen in Figure 3 for nylon-6 (N-6) [58,64].

Consequently, when coalesced-N-6 (c-N-6) is added in small amounts to as-received N-6 (asr-N-6) the resulting sample (nuc-N-6) crystallizes in a manner similar to neat c-N-6. Such self-nucleated polymers can be effectively used as reinforcement in single-component polymer composites [64], and will be discussed later.

### Coalesced amorphous polymers

**Figure 4:**
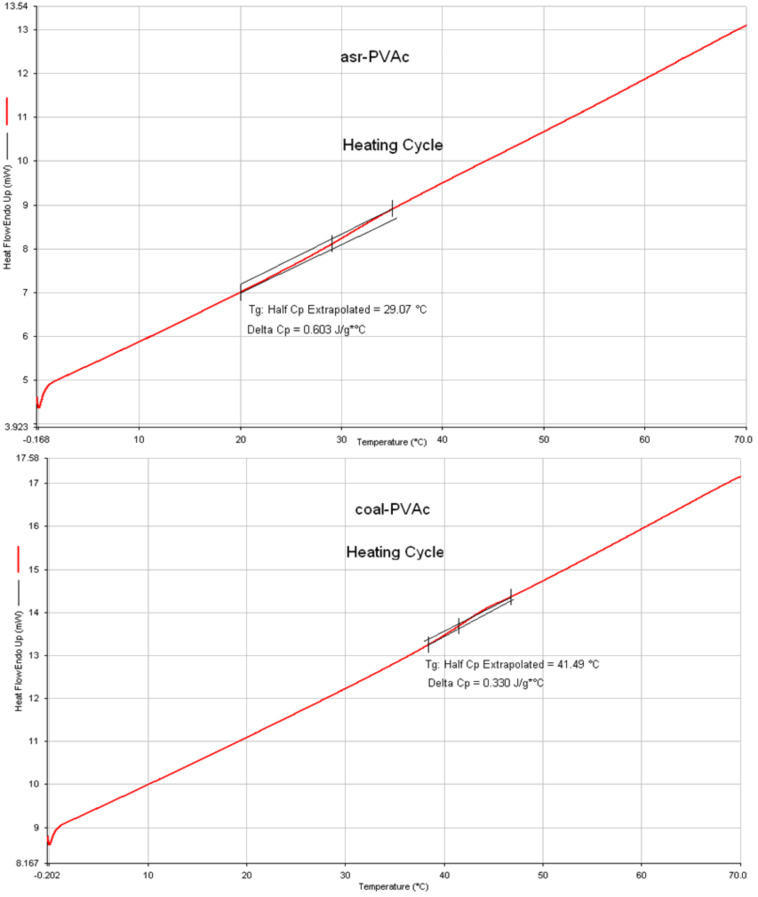
DSC heating scans for asr-PVAc (upper) and c-PVAc coalesced from its γ-CD IC (lower) [72].

We begin describing the behaviors of amorphous polymers coalesced from their CD ICs, with atactic poly(vinyl acetate) (PVAc) as an example [44,71]. In Figure 4 we can see that the *T*_g_ of c-PVAc is more than 12 °C higher than that of asr-PVAc, an observation typical of amorphous polymers coalesced from their CD ICs [42]. Table 1 presents the densities of both PVAc samples measured [71] below and above their *T*_g_’s. The higher *T*_g_ of c-PVAc is consistent with its higher density, which remains higher than that of asr-PVAc even after being annealed at well above their *T*_g_’s, at 70 °C, for several weeks.

**Table 1:** Measured densities for as-received and coalesced PVAcs [71].

sample	density at 25 °C (g/cm^3^) (below *T*_g_)	density at 58 °C (g/cm^3^) (above *T*_g_)

asr-PVAc	1.093	1.040
c-PVAc	1.156	1.077

The high-temperature stability of the reorganized structures and resultant behaviors of c-polymers, as typified here by c-PVAc, has been repeatedly observed [17,52,63,71] and will be revisited and discussed further after we have completed our presentation of c-polymer behaviors. Also we have recently observed that PVAc coalesced from its IC formed with host urea (U) behaves quite similarly to PVAc coalesced from its γ-CD IC [72].

### Coalesced semicrystalline polymers

**Figure 5:**
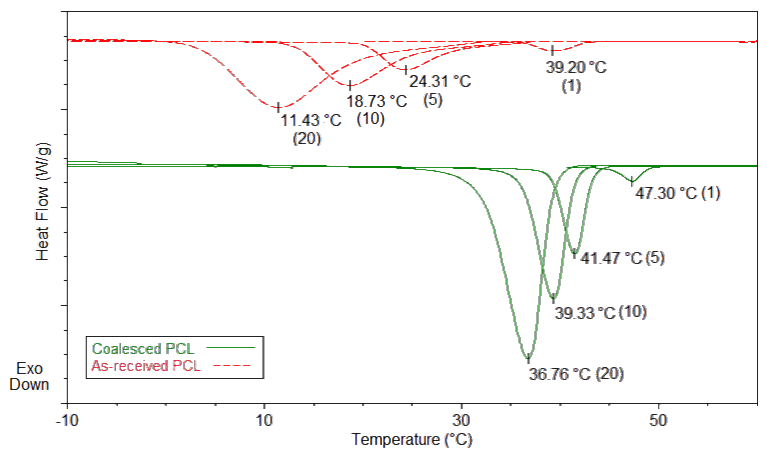
Melt-crystallization curves of as-received and coalesced PCL observed at 20, 10, 5, and 1 °C/min cooling rates [65].

Poly(ε-caprolactone) (PCL) is a biodegradable/bioabsorbable aliphatic polyester that is often used in biomedical applications, such as drug delivery and suture manufacturing. However, its relatively poor physical properties limit its use in load-bearing applications. An attempt to improve the strength of PCL was made by processing with α-cyclodextrin (α-CD). First an inclusion complex (IC) between PCL and α-CD was formed, and then the host α-CD was stripped away to yield bulk coalesced PCL (c-PCL), a process referred to as coalescence [65]. The thermal, physical, and melt rheological properties of c-PCL resulting from this coalescence process were observed to be improved, as a result of the largely extended, unentangled coalesced PCL chains. This also resulted in substantial increases in melt-crystallization temperatures, *T*_c_’s (up to 25 °C higher, depending on the cooling rate from the melt), as illustrated in Figure 5, even though PCL is ordinarily an inherently “fast melt-crystallizer”. Similarly enhanced crystallizabilities were also observed for PCLs of various molecular weights when coalesced from their U ICs [68].

Density and DSC measurements [70] revealed a closer packing of chains in the noncrystalline sample regions, but this did not affect the overall crystallinity of the c-PCL films. Increased elastic storage modulus, decreased tan δ, increased average hardness (33%), and increased Young’s modulus (53%) [65] were observed for the c-PCL films. Annealing c-PCL well above *T*_m_ (90 °C) for a month, did not cause the reorganized c-PCL chains in the noncrystalline regions to revert to the normal randomly coiled entangled melt. This permitted c-PCL to be used as a homogeneous nucleant in the melt-crystallization of as-received PCL (asr-PCL), because when a few percent of c-PCL was added to asr-PCL its melt-crystallization was also found to be accelerated. Thus, by means of melt-processing with c-PCL added as a nucleant, the semi-crystalline morphology of PCL may be controlled. Of course, not only is the c-PCL nucleant necessarily nontoxic and biodegradable/bioabsorbable, it is also chemically compatible and has a “stealthy” nature.

Bulk, as-received poly(ethylene terephthalate) (asr-PET) has been observed to reorganize both morphologically and conformationally, either by formation of a crystalline inclusion complex (IC) between guest PET and host γ-cyclodextrin (γ-CD), followed by removal of the host γ-CD and coalescence of the guest PET (c-PET), or by precipitation (p-PET) from its solution in trifluoroacetic acid upon gradual addition to a large excess of rapidly stirred acetone [17,52,69]. The c- and p-PETs showed very similar behaviors, but p-PET can be more easily produced in larger quantities. DSC and density observations of p-PET imply structures/morphologies and chain conformations and packing in the noncrystalline sample regions that are different from those of asr-PET obtained by standard processing techniques.

In comparison to slowly crystallizing/easily melt-quenched asr-PET, p-PET repeatedly crystallizes rapidly from the melt. Upon subsequent heating, its noncrystalline domains do not show a glass transition or undergo crystallization, but only a melting endotherm that is virtually identical in magnitude to the crystallization exotherm observed during its prior rapid cooling from the melt, is observed (see DSC results and further discussion of p-PET below). These observations suggest that p-PET readily attains higher crystallinity even when repeatedly cooled rapidly from the melt. Apparently the extended conformations of largely unentangled chains in p-PET do not become coiled and entangled even after spending substantial time in the melt.

As a consequence, we have demonstrated [69] that p-PET can be used in small quantities (a few percent) as an effective self-nucleating agent to control the bulk semicrystalline morphology of melt-processed asr-PET, and the resulting properties of nucleated PET (nuc-PET) were assessed. For instance, comparison of asr- and nuc-PET films, each with ~10% crystallinity, reveals that the nuc-PET film has significantly increased density, hardness and Young’s modulus and is also much less permeable to CO_2_ than the asr-PET film. Undrawn nuc-PET fibers also exhibited significantly higher tenacities and moduli than undrawn asr-PET fibers. Self-nucleated PET not only possesses improved properties, but contains no incompatible additives, and so may be readily recycled.

### Coalesced block copolymers

The triblock copolymer PCL-PPG-PCL, with noncrystallizable central poly(propylene oxide blocks), was synthesized by coordinated ring-opening polymerization of ε-caprolactone with PPG-diol as the initiator [21]. In the IC of PCL-PPG-PCL formed with α-CD, only PCL blocks were included. In contrast, both PCL and PPG blocks were included in the IC of PCL-PPG-PCL formed with γ-CD, which has larger channels. Consequently, coalescence of the triblock copolymer chains from these two CD ICs yielded samples showing opposite changes in the segregation and crystallinity (*X*_c_) of the PCL blocks.

As can be seen in Table 2, the crystallinity of the sample coalesced from the triblock-α-CD IC is obviously higher than that of the as-synthesized triblock copolymer. On the contrary, the crystallinity of the sample coalesced from the γ*-*CD IC is lower than that of the as-synthesized triblock copolymer [21]. This difference is a result of the fact that the entire triblock is included in the crystalline channels of the γ*-*CD IC, while only the PCL blocks are included in the α-CD IC (Figure 1).

**Table 2:** Thermal properties and crystallinities of various PCL-PPG-PCL triblock copolymer samples, as revealed by DSC [21].

sample	*T*_m_ (°C)	Δ*H*_m_ (J/g)	*Χ*_c_ (%)

as-synthesized copolymer	57.3	58.6	56.5
coalesced from α-CD IC	63.8	76.8	74.1
coalesced from γ-CD IC	63.0	51.3	49.5

As a result, the segregation or mixing of PCL and PPO blocks in their CD-ICs is carried over to the coalesced tri-block copolymer samples, explaining the divergent crystallinities of the PCL blocks.

The fact that the PCL-PPG-PCL triblock coalesced from its γ*-*CD IC is not dramatically less crystalline than the fully segregated as-synthesized sample, is likely a result of the ability of two PCL blocks to occupy the same γ*-*CD IC channels [73], which are larger and, consequently, lead to partial segregation of the crystallizable PCL blocks.

When PCL-*b*-PLLA [poly(L-lactic acid)] was obtained by first forming its IC with α-CD, followed by coalescence of the guest diblock copolymer chains, a readily biodegradable sample of the block copolymer with very low crystallinity was produced [16]. Compression molding between Teflon plates produced film samples of asr- and c-PCL-*b*-PLLA, PCL and PLLA homopolymers of approximately the same chain lengths as the corresponding blocks in PCL-*b*-PLLA, and a physical blend of PCL/PLLA homopolymers with the same molar composition as the PCL-*b*-PLLA. The in vitro biodegradation behaviors of these films were observed in phosphate buffer solution containing lipase from *Rhizopus arrhizus* by means of ultraviolet and attenuated total reflectance FTIR spectroscopy, DSC, wide-angle X-ray diffraction, and weight-loss analysis.

**Figure 6:**
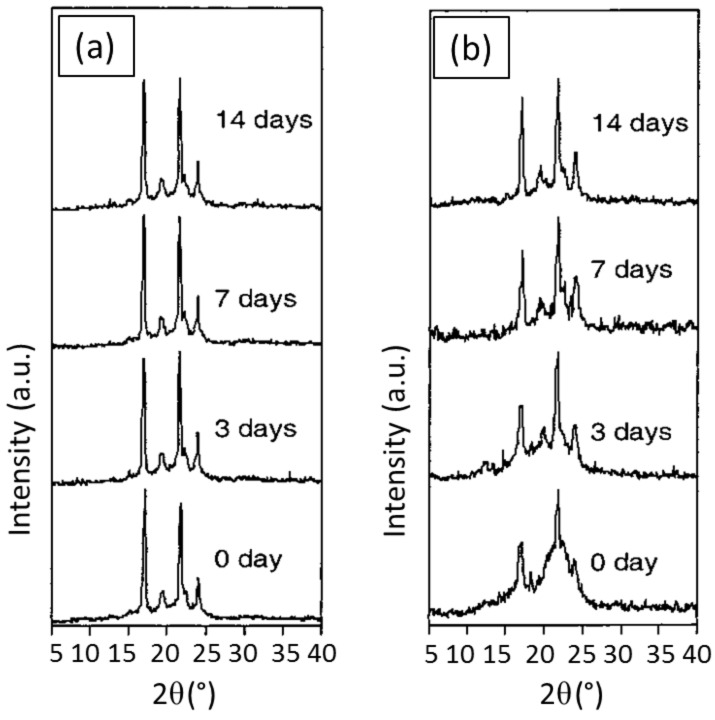
X-ray diffraction patterns of as-synthesized PCL-*b*-PLLA films (a) and coalesced PCL-*b*-PLLA films (b), after various enzymatic degradation times [16,25].

The PCL segments in all of the above films were found to degrade much faster than the PLLA segments. As expected, suppression of the phase segregation that resulted from mixing of PCL and PLLA blocks leading to decreased crystallinity in the c-diblock copolymer film, resulted in a much faster enzymatic degradation than that of either the asr-diblock copolymer or the PCL/PLLA physical blend. The biodegradation of the c-diblock was observed to be especially enhanced during the early stages. The disappearance of amorphous scattering and a sharpening of the crystalline peaks in the X-ray diffractograms seen in Figure 6 make clear that it is the well-mixed amorphous portions of the c-PCL-*b*-PLLA diblock copolymer film that are preferentially degraded by the enzyme. Regulation of their biodegradation behavior, through formation of and coalescence from CD ICs, may enhance the use of block copolymers in drug delivery and controlled release systems, because of its decisive importance in these applications.

### Coalesced polymer blends

**Figure 7:**
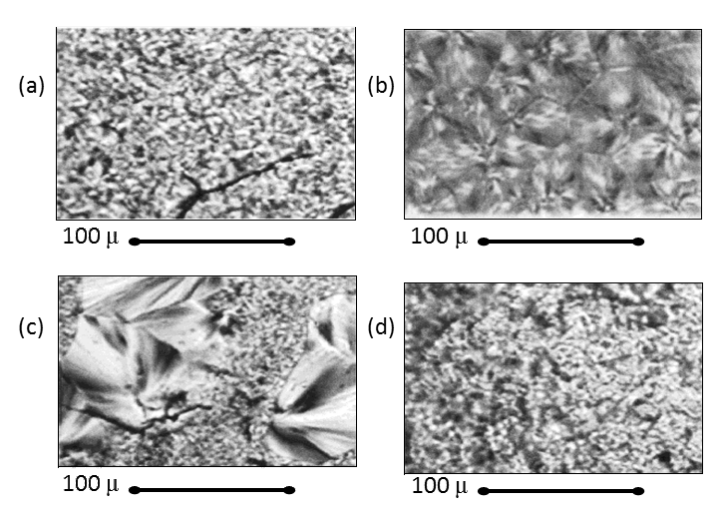
Polarizing photomicrographs of (a) PLLA, (b) PCL, (c) solution-cast, and (d) coalesced PLLA/PCL blends [8].

**Figure 8:**
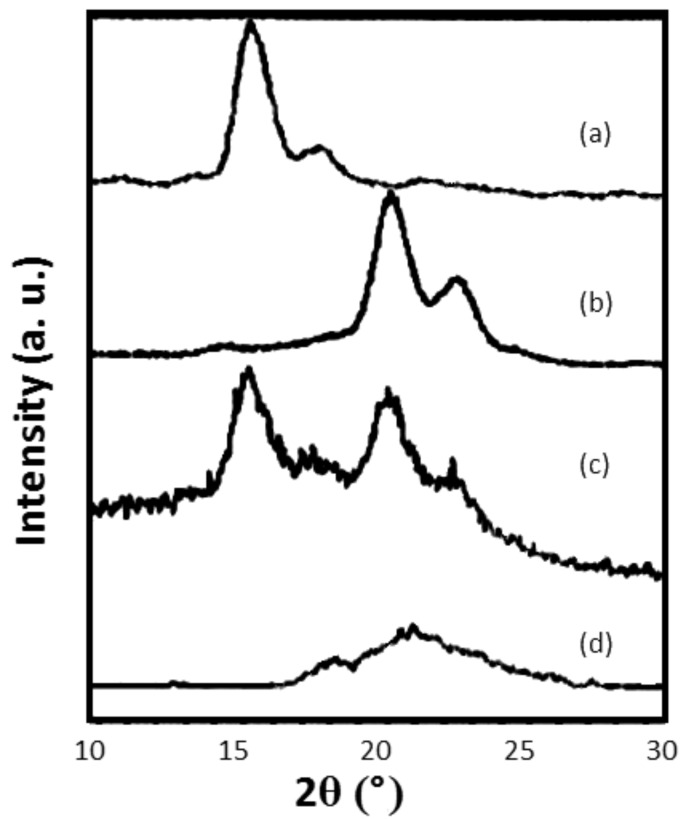
X-ray diffractograms of (a) pure PCL and (b) PLLA and PCL/PLLA blends obtained by casting from dioxane solution (c) and hot-water coalescence from PCL/PLLA–CD IC (d) [16,25].

Intimately mixed PCL/PLLA blends were obtained upon coalescence from their common α-CD IC [7], as suggested by the polarized micrographs and X-ray diffractograms shown in Figure 7 and Figure 8, respectively. Two-dimensional spin-diffusion NMR observations [40] of these blends demonstrated that individual PCL and PLLA chains are indeed in intimate contact.

**Figure 9:**
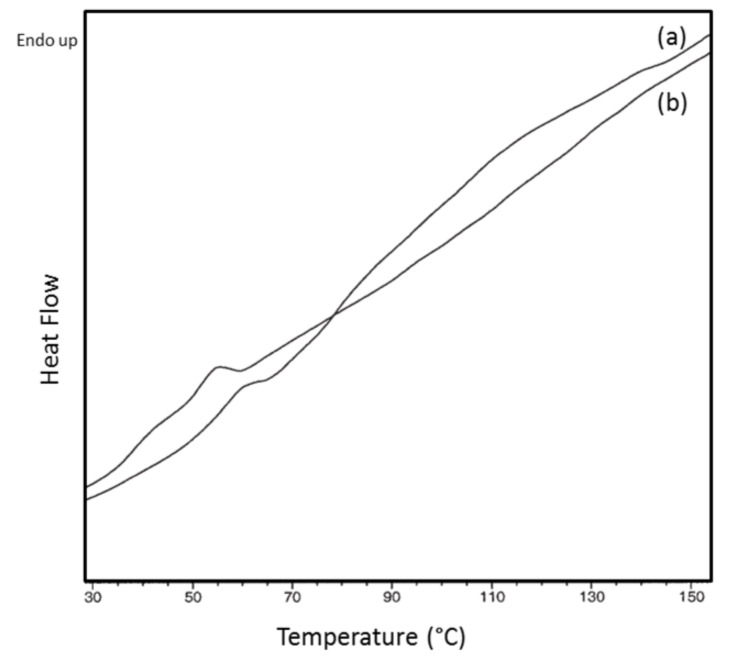
MDSC scans of the (a) first and (b) second heating runs recorded for the PC/PMMA/PVAc-2 blend. The sample was held for 3 min at 170 °C after the first heating [28].

A ternary PVAc/poly(methyl methacrylate) (PMMA)/polycarbonate (PC) blend was coalesced from their common IC formed with host γ*-*CD [28]. Intimate mixing of all three polymers was observed, as indicated by the single *T*_g_ exhibited in DSC observations of their ternary coalesced blend (Figure 9). Solid-state NMR observations [^13^C observed ^1^H spin-lattice relaxation times recorded in the rotating frame, *T*_1ρ_(^1^H)] of the three polymers in their ternary blend confirmed their intimate molecular mixing on a scale less than 5 nm.

### Thermal stability of coalesced polymer structures and behaviors

As noted in passing above, in our discussion of coalesced polymers both amorphous and crystallizable, the many unusual behaviors and properties they exhibit are stable to long periods of high-temperature annealing, above their *T*_g_’s and *T*_m_’s [57,74]. These observations suggest solid-state organizations/structures/morphologies for coalesced polymers that are distinct from those that are normally processed from their solutions and melts. Furthermore, their stability to long periods of high-temperature annealing also indicates that their melts are and remain distinct from those samples that are processed normally.

**Figure 10:**
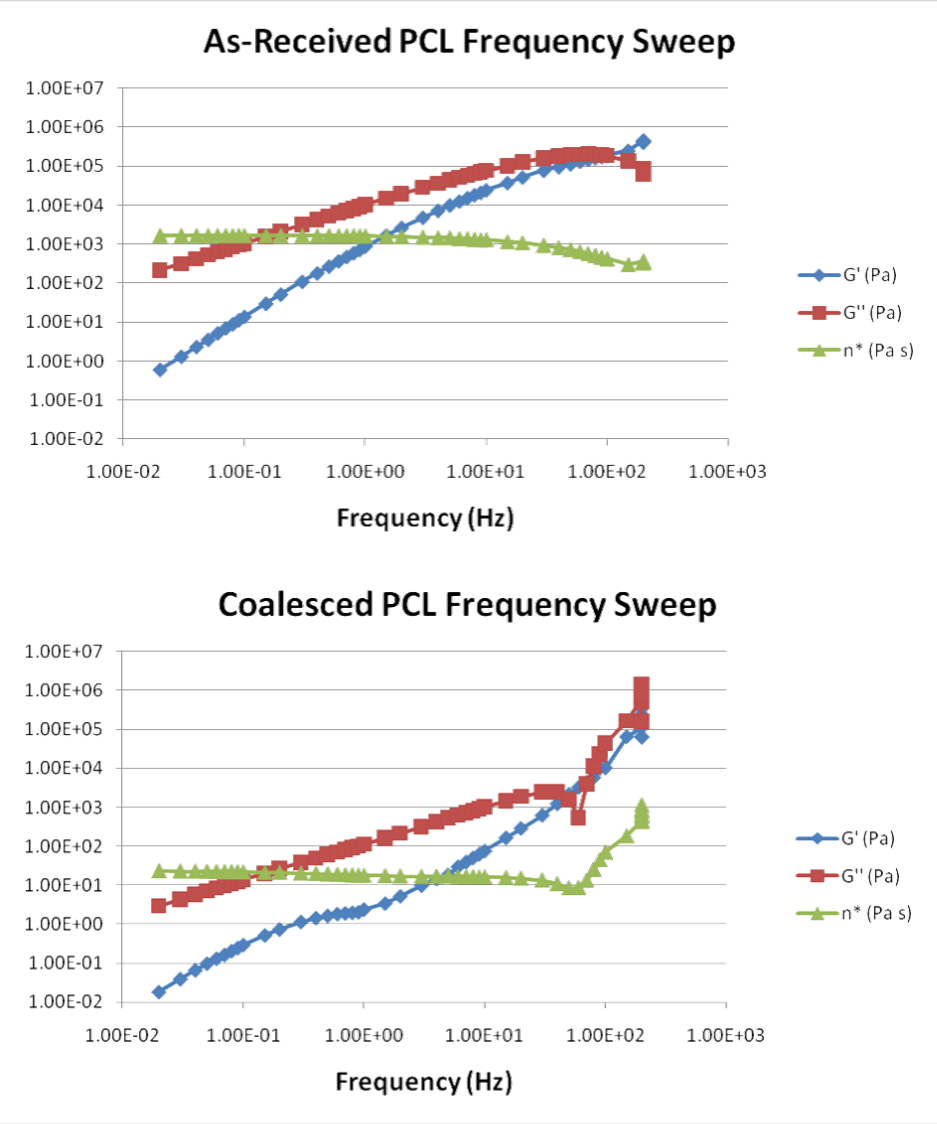
Storage modulus, loss modulus, and apparent viscosity (G’, G’’, and n*, respectively) for asr- and c-PCL melts, (top and bottom, respectively), as obtained through oscillatory melt rheology (*T* = 90 °C; testing stress = 750 Pa; pretest hold time = 1 minute) [65,70].

For example, in Figure 10 the rheological behaviors of asr- and c-PCL melts, the latter obtained from PCL-α-CD IC, are compared and seen to be quite distinct [65,70]. The zero shear viscosity of the c-PCL melt is about two orders of magnitude less than that of the asr-PCL melt. Repetitive rheological runs on the same asr- and c-PCL samples demonstrated that the distinct rheological responses of their melts were independent of long-time melt annealing, as well as long exposures to rheological stresses.

So, what are the organizations/structures in c-polymer melts?

What we do know [2,57,65,70,74]:

1. They behave differently: (a) They crystallize more readily and apparently without chain folding. (b) They have elevated *T*_g_’s and *T*_c_’s. (c) Their blends are intimately mixed. (d) Their amorphous regions are denser. (e) Their melts have much lower zero shear viscosities. (f) They produce stronger, less extensible films and fibers. (g) They are less permeable to gases (CO_2_).
2. All the above behaviors remain, even after extensive periods (weeks) spent in their melts [57,65,68–69,72,74] and, though only mentioned very briefly here, are independent of their molecular weights and their IC host (CD or U).
3. We anticipate that upon coalescence from their ICs, the resulting coalesced polymer samples will consist of small disoriented regions (smaller than the sizes of their IC crystals) of extended, unentangled, and oriented chains (Figure 1), because polymer ICs are generally obtained as crystalline powders. That is not to say that the initial overall macroscopic orientation of all extended and unentangled chains is a result of this. Instead, the macroscopic organization of polymer chains in the melt may initially resemble a grouping of small, randomly arranged “nematic-like” regions, i.e., without a preferred orientation of their directors. For a discussion of Vectra, a liquid-crystalline ester/arylate copolymer, which exhibits a macroscopically anisotropic melt, much like that suggested above locally for coalesced polymers and with similar rheological behavior (Beers and Ramirez [75]).
4. This anticipated structure is consistent with their behaviors noted in point 1, including their melt rheologies. Though we have discussed potential reasons for the long-time, high-temperature stability noted in point 2 [57,74], we have yet to connect it to the structures of coalesced polymer samples.

So the question remains, how can we directly observe the structure(s) of coalesced polymers in their melts? To date we have been unable to answer this question, and, so, invite the reader to offer suggestions.

### Coalesced polymer applications: Scientific and commercial

Our brief discussion above concerning the behaviors of c- and p-PETs, and how they may be used to self-nucleate the melt-crystallization of asr-PET to produce nuc-PET materials with improved properties, may not only have commercial significance, but may in addition enhance our understanding of the underlying bases for polymer structure–property relations in PET and other polymers.

**Figure 11:**
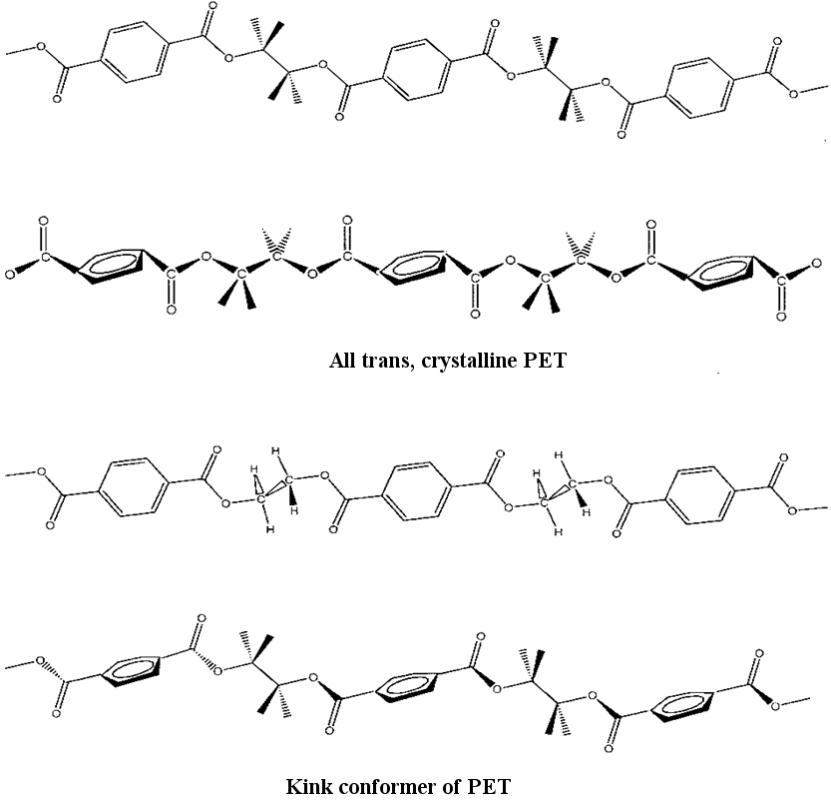
Crystalline all *trans* (*t*) and γ-CD-included *g±tg*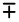 conformations of PET [76].

**Figure 12:**
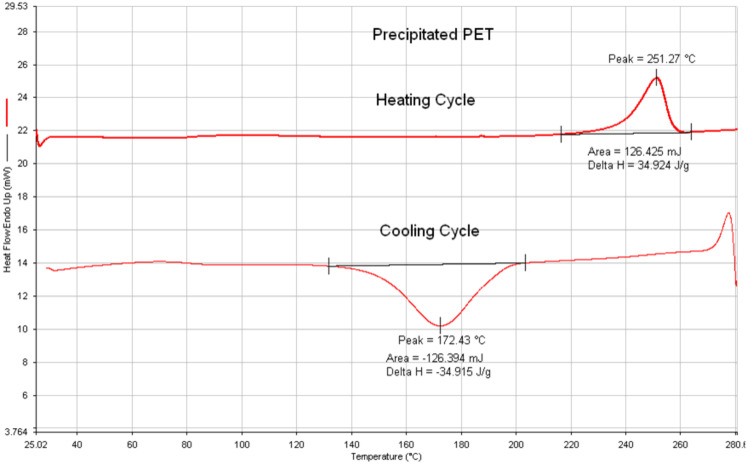
DSC scans for p-PET [70].

As described previously [17,52], γ-CDs are capable of forming an inclusion compound with PET. Modeling of PET conformations able to thread through CDs suggested that the *gauche*± *trans gauche*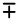 ethylene glycol conformations illustrated in Figure 11 have a narrower cross section than the all trans crystalline PET conformation also illustrated there [76]. Analyses of the FTIR [17] and solid-state ^13^C NMR [27] spectra of PET coalesced from its γ-CD IC (c-PET) are consistent with the narrower *gauche± trans gauche*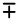 conformations for the non-crystalline portions of the coalesced sample. Unlike normal as-received PET (asr-PET), c-PET was observed to be repeatedly rapidly crystallizable from its melt. During the course of forming the PET-γ-CD IC [17], several control experiments were conducted that led to the observation that PET that was slowly precipitated (p-PET) from TFA solution with rapidly stirred acetone, exhibited thermal behavior very similar to that of c-PET [52,69], which can be seen in Figure 12.

It has been suggested [17,52,69] that the chains of c- and p-PETs in their noncrystalline regions largely adopt the extended *gauche± trans gauche*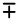 conformations, with *trans* –CH_2_–CH_2_– bonds, as in their crystals. Normally in the melt, the –CH_2_–CH_2_– bond is predominantly *gauche±* [77–78], and so must rotate to *trans* during crystallization. This conformational transition is not possible without sweeping out a large volume. On the other hand, crystallization of c- or p-PETs into the all-trans conformation proceeds rapidly from preponderantly *gauche±* –O–CH_2_–, *trans* –CH_2_–CH_2_– and *gauche*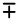 –CH_2_–O– bond conformers through facile counter rotations about the –O–CH_2_– and –CH_2_–O– bonds, requiring only a very modest amount of swept-out volume [17,52,69]. Thus, it may not be surprising that asr-PET crystallizes slowly from its melt, while c- and p-PETS crystallize rapidly.

Quenched asr- and nuc-PET films (5 wt % p-PET/95 wt % asr-PET) are clear in appearance. DSC scans of the two films are not shown here, but indicate [69] that both PET films have the same level of crystallinity (~10%). Their densities obtained by using the flotation technique are summarized in Table 3 [69,71]. The higher density of the nuc-PET film (~1.3% higher) with the same low level of crystallinity as in the asr-PET film can likely be attributed to the higher orientation and increased order and packing of its extended unentangled chains in its predominant amorphous domains, which is seen even after the polymer film was quenched from the melt into ice water. This shows that nuc-PET has a tendency to organize differently to asr-PET, even when the melt is quenched at very high cooling rates.

**Table 3:** Densities of asr-PET and nuc-PET [69].

sample	density at 25 °C (g/cm^3^)

asr-PET	1.368
nuc-PET	1.386

As clearly demonstrated in the case of PET, much can be learned about polymer structure–property relationships by forming polymer ICs, coalescing the guest polymers, and observing and comparing their behaviors and properties to those of samples normally processed from their solutions and melts.

**Figure 13:**
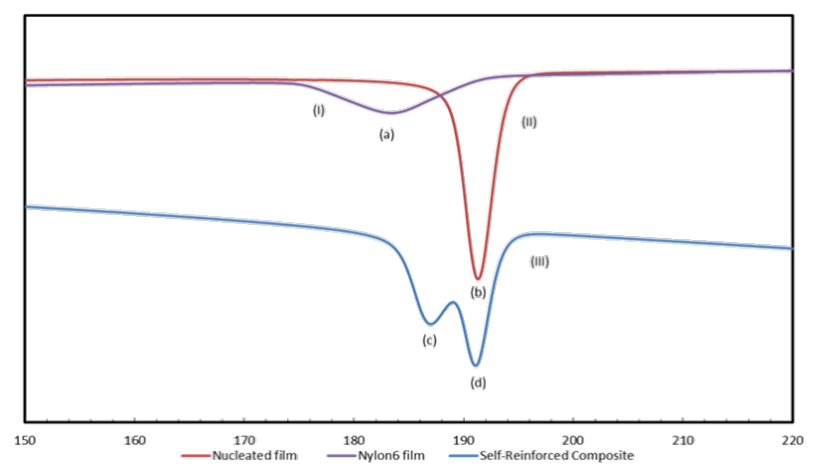
DSC cooling scans from the melts of (I) asr-N-6, (II) nuc-N-6, and (III) asr/nuc N-6 film sandwich. Melt-crystallization peaks (a), (b), (c), and (d) in the DSC scans correspond to *T*_c_ = 183, 192, 186, and 191 °C, respectively [64].

**Figure 14:**
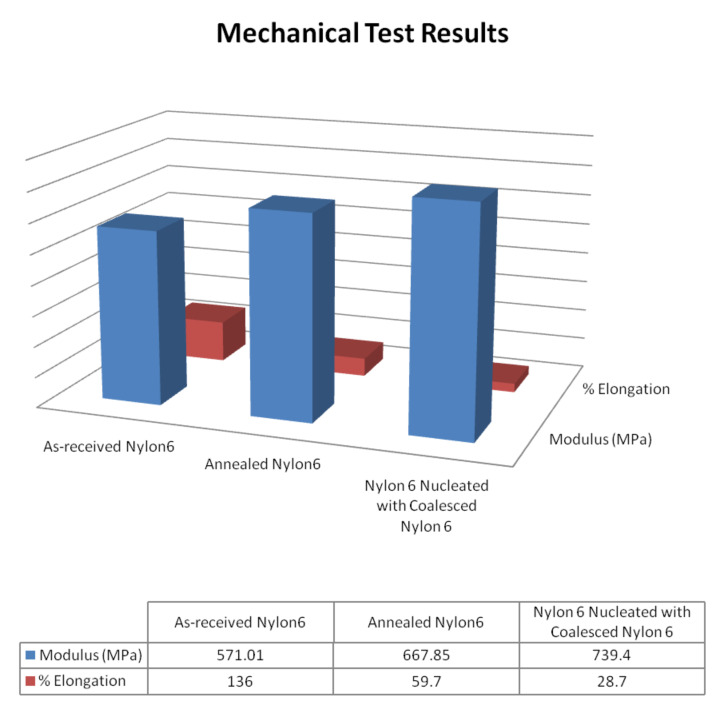
Mechanical properties of N-6 films [62].

Previously in Figure 3, we demonstrated that N-6 coalesced from its α-CD IC (c-N-6) crystallizes more readily. When 2 wt % c-N-6 is added to 98 wt % asr-N-6 to produce nuc-N-6, we similarly observe the nuc-N-6 to crystallize rapidly (Figure 13) [64]. This leads to improved mechanical properties for nuc-N-6, as illustrated in Figure 14, in which asr-N-6 film has also been annealed to exhibit very similar crystallinity to the nuc-N-6 film.

**Figure 15:**
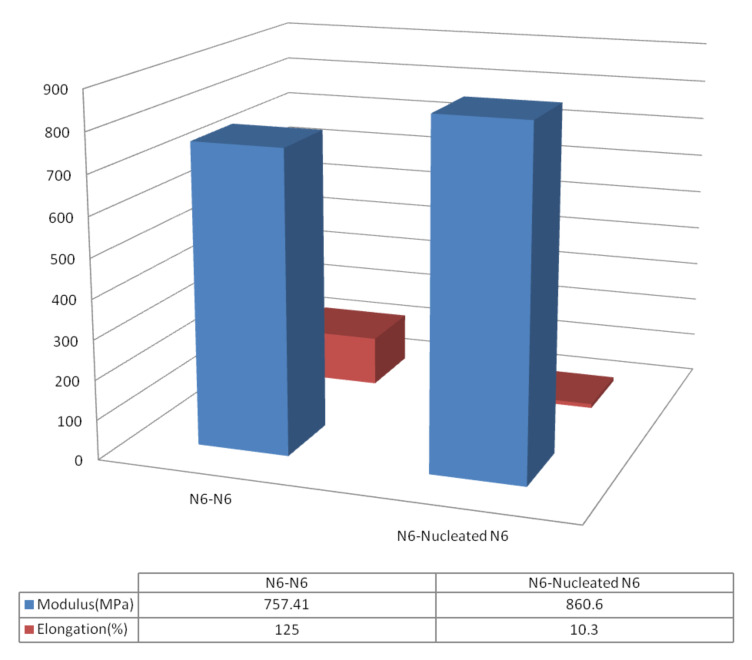
Tensile testing of as-received/as-received and as-received/nucleated nylon-6 film sandwiches conducted according to ASTM D-882-97. Each value of the mechanical properties reported is an average of at least five film-sandwich specimens [64].

As a consequence, we created film sandwiches formed with two layers of asr-N-6 and a composite sandwich with one layer each of asr- and nuc-N-6 films. Note in Figure 13 the composite N-6 sandwich retains distinct thermal responses for each of its constituent layers despite ~10 min of melt processing. The mechanical properties of the asr-N6/asr-N-6 control and asr-N-6/nuc-N-6 composite sandwiches are presented in Figure 15. Stress-strain observations (not shown) of both film sandwiches reveal, unsurprisingly, very strong interfaces for both sandwiches [64].

This work has been extended to melt-spun N-6 fibers, with similar results, confirming that c-polymers can serve to nucleate the melt-crystallization of chemically identical polymers, resulting in improved mechanical properties for the nuc-polymers. Such nuc-polymers can be effectively used as reinforcements in single-component composites, which necessarily have strong interfaces, and which show improved properties in comparison to the asr-polymer matrices.

## Conclusion

We have attempted to demonstrate the utility of restructuring polymers, through nanoconfinement in and subsequent release/coalescence from their noncovalently bonded inclusion compounds, for the reorganization of their resulting bulk samples, thereby improving their properties. Because c-polymers remain reorganized despite long-term melt-annealing, they may be successfully melt-processed into improved materials. The examples of coalesced polymers discussed here were all obtained from their CD ICs. However, we have recently observed [68,72] very similar behavior for polymers coalesced from their U ICs, for which in this case the host molecules cannot thread over the included polymer chains. We therefore conclude that the unique behaviors of polymer samples coalesced from their CD ICs is not a consequence of remnant host CDs threaded over their chains.

Though mentioned only in passing here, significant information regarding the conformations, mobilities and supramolecular assembly of polymers can be obtained by observing and modeling both the formation of polymer ICs [47,73,76] and the behaviors of their included, highly extended, and isolated guest polymer chains [73,76]. Finally, we have also tried to indicate that examination and modeling of the behaviors of coalesced polymer samples, and their resulting properties, can usefully contribute to our understanding of the bases for structure–property relations observed in polymer materials.

## References

[1] Harada A, Kamachi M (1990). Macromolecules.

[2] Tonelli A E (2009). Adv Polym Sci.

[3] Huang L, Vasanthan N, Tonelli A E (1997). J Appl Polym Sci.

[4] Huang L, Allen E G, Tonelli A E, Pandalai S G (1997). Recent Research Developments in Macromolecular Research.

[5] Huang L, Tonelli A E (1998). J Macromol Sci, Revs Macromol Chem Phys.

[6] Huang L, Allen E, Tonelli A E (1998). Polymer.

[7] Huang L, Tonelli A E, Dinh S M, DeNuzzio J D, Comfort A R (1999). Inclusion Compounds as a Means to Fabricate Controlled Release Materials. Intelligent Materials for Controlled Release.

[8] Rusa C C, Tonelli A E (2000). Macromolecules.

[9] Huang L, Gerber M, Taylor H, Lu J, Tapaszi E, Wutkowski M, Hill M, Nunalee F N, Harvey A, Rusa C C, Povder T, Urban M W (2001). Creation of Polymer Films with Novel Structures by Processing with Inclusion Compounds. Film Formation in Coatings: Mechanisms, Properties, and Morphology.

[10] Lu J, Mirau P A, Rusa C C, Tonelli A E, Szejtli J (2001). Cyclodextrin: From Basic Research to Market. Proceedings of the 10th International Cyclodextrin Symposium (CD-2000).

[11] Rusa C C, Lu J, Huang L, Tonelli A E, Szejtli J (2001). Cyclodextrin: From Basic research to Market. Proceedings of the 10th International Cyclodextrin Symposium (CD-2000).

[12] Rusa C C, Luca C, Tonelli A E (2001). Macromolecules.

[13] Wei M, Tonelli A E (2001). Macromolecules.

[14] Shuai X, Porbeni F E, Wei M, Shin I D, Tonelli A E (2001). Macromolecules.

[15] Huang L, Gerber M, Taylor H, Lu J, Tapazsi E, Wutkowski M, Hill M, Lewis C, Harvey A, Wei M (2001). Macromol Symp.

[16] Shuai X, Wei M, Porbeni F E, Bullions T A, Tonelli A E (2002). Biomacromolecules.

[17] Bullions T A, Wei M, Porbeni F E, Gerber M J, Peet J, Balik M, White J L, Tonelli A E (2002). J Polym Sci, Part B: Polym Phys.

[18] Shuai X, Porbeni F E, Wei M, Bullions T, Tonelli A E (2002). Macromolecules.

[19] Wei M, Davis W, Urban B, Song Y, Porbeni F E, Wang X, White J L, Balik C M, Rusa C C, Fox J (2002). Macromolecules.

[20] Rusa C C, Bullions T A, Fox J, Porbeni F E, Wang X, Tonelli A E (2002). Langmuir.

[21] Shuai X, Porbeni F E, Wei M, Bullions T, Tonelli A E (2002). Macromolecules.

[22] Wei M, Shuai X, Tonelli A E (2003). Biomacromolecules.

[23] Bullions T A, Edeki E M, Porbeni F E, Wei M, Shuai X, Rusa C C, Tonelli A E (2003). J Polym Sci, Part B: Polym Phys.

[24] Abdala A A, Tonelli A E, Khan S A (2003). Macromolecules.

[25] Tonelli A E (2003). J Tex Appar Tech Mgmt.

[26] Tonelli A E (2003). Macromol Sympos.

[27] Wei M, Bullions T A, Rusa C C, Wang X, Tonelli A E (2004). J Polym Sci, Part B: Polym Phys.

[28] Rusa C C, Uyar T, Rusa M, Hunt M A, Wang X, Tonelli A E (2004). J Polym Sci, Part B: Polym Phys.

[29] Abdala A A, Wu W, Olesen K R, Jenkins R D, Tonelli A E, Khan S (2004). J Rheol.

[30] Wei M, Shin I D, Urban B, Tonelli A E (2004). J Polym Sci, Part B: Polym Phys.

[31] Rusa C C, Shuai X, Bullions T A, Wei M, Porbeni F E, Lu J, Huang L, Fox J, Tonelli A E (2004). J Polym Environ.

[32] Uyar T, Rusa M, Tonelli A E (2004). Makromol Rapid Commun.

[33] Rusa C C, Wei M, Bullions T A, Rusa M, Gomez M A, Porbeni F E, Wang X, Shin I D, Balik C M, White J L (2004). Cryst Growth Des.

[34] Rusa C C, Wei M, Shuai X, Bullions T A, Wang X, Rusa M, Uyar T, Tonelli A E (2004). J Polym Sci, Part B: Polym Phys.

[35] Rusa M, Aboelfotoh O, Kolbas R M, Tonelli A E (2004). PMSE Prepr.

[36] Rusa M, Wang X, Tonelli A E (2004). Macromolecules.

[37] Rusa C C, Wei M, Bullions T A, Shuai X, Uyar T, Tonelli A E (2005). Polym Adv Technol.

[38] Rusa C C, Rusa M, Gomez M, Shin I D, Fox J D, Tonelli A E (2004). Macromolecules.

[39] Hernández R, Rusa M, Rusa C C, López D, Mijangos C, Tonelli A E (2004). Macromolecules.

[40] Jia X, Wang X, Tonelli A E, White J L (2005). Macromolecules.

[41] Uyar T, Rusa C C, Wang X, Rusa M, Hacaloglu J, Tonelli A E (2005). J Polym Sci, Part B: Polym Phys.

[42] Rusa C C, Bridges C, Ha S-W, Tonelli A E (2005). Macromolecules.

[43] Uyar T, Rusa C C, Hunt M A, Aslan E, Hacaloglu J, Tonelli A E (2005). Polymer.

[44] Uyar T, Aslan E, Tonelli A E, Hacaloglu J (2006). Polym Degrad Stab.

[45] Uyar T, Hunt M A, Gracz H S, Tonelli A E (2006). Cryst Growth Des.

[46] Uyar T, Oguz G, Tonelli A E, Hacaloglu J (2006). Polym Degrad Stab.

[47] Rusa C C, Rusa M, Peet J, Uyar T, Fox J, Hunt M A, Wang X, Balik C M, Tonelli A E (2006). J Inclusion Phenom Macrocyclic Chem.

[48] Pang K, Schmidt B, Kotek R, Tonelli A E (2006). J Appl Polym Sci.

[49] Uyar T, Gracz H S, Rusa M, Shin I D, El-Shafei A, Tonelli A E (2006). Polymer.

[50] Uyar T, Tonelli A E, Hacaloğlu J (2006). Polym Degrad Stab.

[51] Tonelli A E, Brown P, Stevens K (2007). Nanofibers and Nanotechnology in Textiles.

[52] Vedula J, Tonelli A E (2007). J Polym Sci, Part B: Polym Phys.

[53] Uyar T, Rusa C C, Tonelli A E, Hacaloğlu J (2007). Polym Degrad Stab.

[54] Martínez G, Gómez M A, Villar-Rodil S, Garrido L, Tonelli A E, Balik C M (2007). J Polym Sci, Part A: Polym Chem.

[55] Tonelli A E (2008). J Inclusion Phenom Macrocyclic Chem.

[56] Tonelli A E (2008). Polymer.

[57] Tonelli A E (2009). J Polym Sci, Part B: Polym Phys.

[58] Mohan A, Joyner X, Kotek R, Tonelli A E (2009). Macromolecules.

[59] Busche B J, Tonelli A E, Balik C M (2010). Polymer.

[60] Busche B J, Tonelli A E, Balik C M (2010). Polymer.

[61] Busche B J, Tonelli A E, Balik C M (2010). Polymer.

[62] Mohan A, Gurarslan A, Joyner X, Child R, Tonelli A E (2011). Polymer.

[63] Williamson B R, Tonelli A E (2012). J Inclusion Phenom Macrocyclic Chem.

[64] Gurarslan A, Tonelli A E (2011). Macromolecules.

[65] Williamson B R, Krishnaswany R, Tonelli A E (2011). Polymer.

[66] Peet J, Rusa C C, Hunt M A, Tonelli A E, Balik C M (2005). Macromolecules.

[67] Rusa C C, Fox J, Tonelli A E (2003). Macromolecules.

[68] Gurarslan A, Shen J, Tonelli A E (2012). Macromolecules.

[69] Joijode A S, Hawkins K, Tonelli A E (2012). Polymer.

[70] Williamson B R (2010).

[R71] 71The densities of asr- and c-PVAc samples were measured as described in [65] by floatation using water and aq NaBr (21 wt %) (densities of 1.0 and 1.184 g/cm^3^, respectively, lower and higher than that of PVAc). Into a known volume of water, vol(H_2_O), containing a magnetic stirring bar, were placed small pieces of both PVAc films pressed at 70 °C, which sank to the bottom. The NaBr/H_2_O solution was slowly added from a burette, under stirring, until each PVAc film in turn rose from the bottom and was suspended in the aq solution, and the volume of added NaBr/H_2_O, vol(NaBr/H_2_O), was noted. The densities of asr- and c-PVAc films were then obtained as 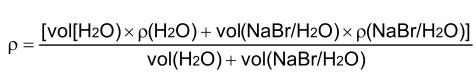 both below and above their glass-transition temperatures.

[72] Joijode A S, Gurarslan A, Tonelli A E (2012). Macromolecules.

[73] Lu J, Mirau P A, Tonelli A E (2002). Prog Polym Sci.

[74] Gurarslan A, Joijode A S, Tonelli A E (2012). J Polym Sci, Part B: Polym Phys.

[75] Beers D E, Ramirez J E (1990). J Text Inst.

[76] Tonelli A E (1992). Comput Theor Polym Sci.

[77] Williams A D, Flory P J (1967). J Polym Sci, Part A-2.

[78] Kaji H, Schmidt-Rohr K (2002). Macromolecules.

